# Comparative Analysis of Pre-Gelatinized Potato Starch Prepared by Different Drying Methods and Its Influence on Noodle Quality

**DOI:** 10.3390/foods15203752

**Published:** 2026-10-21

**Authors:** Fen Xu, Chenxing Wang, Hanyi Duan, Lingyi Li, Jianfu Liu, Honghai Hu, Christophe Blecker

**Affiliations:** 1 Tianjin Key Laboratory of Food and Biotechnology, School of Biotechnology and Food Science, Tianjin University of Commerce, Tianjin 300134, China; 2 Tianjin International Joint Research and Development Center for Food Science and Engineering, Tianjin 300134, China; 3 Institute of Food Science and Technology, Chinese Academy of Agricultural Sciences (CAAS), Integrated Laboratory of Potato Staple Food Processing Technology of the Ministry of Agriculture and Rural Affairs, Key Laboratory of Agricultural Product Processing and Storage of the Ministry of Agriculture and Rural Affairs, Beijing 100193, China; 4 Department of Food Science and Formulation, Gembloux Agro-Bio Tech, Université de Liège, Passage des Déportés 2, B-5030 Gembloux, Belgium

**Keywords:** crystalline structure, freeze drying, microstructure, oven drying, retrogradation, starch gelatinization

## Abstract

The present study investigated the effects of degree of starch gelatinization (DSG) and drying methods (FD: freeze-drying; OD: oven-drying) on the multi-scale structure of potato starch and its functionality in noodles. Potato starches with varying DSG (0–91.39%) were prepared by FD and OD and characterized by DSC, XRD, FTIR, RVA, and SEM. The short- and long-range ordered structure decreased significantly as DSG increased. Notably, OD samples showed significantly higher relative crystallinity than FD samples (18.55% vs. 10.90%). Peak, trough, and final viscosities increased, while breakdown decreased with increasing DSG. Microstructurally, FD samples exhibited fragmented flakes, whereas OD samples formed a dense and compacted solid matrix. In noodles, low-DSG FD starch significantly enhanced lightness (L* increased from 75.27 to 83.27), hardness (increased by 15.9%), gumminess, and chewiness, whereas this hardness-enhancing effect was reversed at higher DSG levels. Strong correlations were observed between DSG and structural parameters (r > 0.80). These findings demonstrate that the drying method fundamentally alters the molecular structure of partially gelatinized starch even at comparable DSG levels, and low-DSG FD starch enhanced noodle color and texture. The findings provide a scientific basis for the targeted selection of drying methods to tailor starch functionality for specific noodle applications.

## 1. Introduction

Potato starch has been extensively used in the food industry due to its unique properties such as thickening, gelling, and stabilizing capabilities [1]. It is widely used in various food products, including soups, sauces, bread, and noodles, owing to its excellent texture, stability, and sensory attributes [2]. Its functionality is intrinsically linked to its multi-scale structure, ranging from the molecular double-helix arrangement to the granular morphology. Native potato starch granules, however, often exhibit limitations such as low cold-water solubility, poor process tolerance, and uncontrollable retrogradation, which restrict their application. To overcome these drawbacks, physical modification methods, particularly pre-gelatinization, have gained significant attention for their label-friendly nature and efficiency in enhancing potato starch functionality [3,4].

Pre-gelatinization involves the disruption of the native crystalline structure of starch using heat and moisture, imparting instant cold-water viscosity and altering its pasting and gelling behaviors [5]. The properties of the resulting pre-gelatinized starch are predominantly governed by two key factors: the degree of starch gelatinization (DSG), which dictates the extent of molecular disordering, and the subsequent drying method, which critically determines the final solid-state structure. While the influence of DSG on starch physicochemical and structural properties has been relatively well-explored [6,7,8], the application of partially gelatinized starch with varying DSG in food systems remains largely unknown, and the role of the drying process remains insufficiently understood. Starch retrogradation is a re-association process of gelatinized starch, which is particularly relevant to the structural and physicochemical changes occurring in pre-gelatinized starch during drying and subsequent processing. In the context of pre-gelatinized starch, retrogradation during drying can significantly impact the final structure and functionality. Different drying techniques, such as freeze-drying (FD) and oven-drying (OD), impose distinct thermal and temporal stresses on the gelatinized starch matrix. FD preserves the instantaneously gelatinized starch structure but is associated with high costs, whereas OD is a lower-cost convective process that may facilitate molecular rearrangements such as retrogradation during prolonged drying. However, it remains unclear whether these two drying methods can lead to fundamentally different structural and functional properties of pre-gelatinized starch even at comparable DSG levels, and how such differences translate into the quality of starch-based composite foods. Noodles are a staple food worldwide and represent an ideal model system for studying starch functionality in a complex food matrix. Their quality attributes, including color and cooking texture, as well as the gluten network formation, are highly dependent on the physicochemical properties of the constituent starch [9,10,11]. Previously, native and pre-gelatinized starch have been extensively applied in the food industry for favorable texture and smoothness of cooked noodles [9,12], and the pre-gelatinized starch employed in these applications has typically been fully gelatinized. However, full gelatinization requires higher temperatures and longer processing times, resulting in higher energy consumption. It is hypothesized that (1) partially gelatinized starch with a moderate DSG may be sufficient, or even advantageous, for enhancing noodle quality; and (2) the drying method will further modulate the structural and functional properties of the pre-gelatinized starch, even at comparable DSG levels, thereby imparting unique qualities to starch-based foods like noodles. The novelty of this work lies in establishing quantitative structure–function relationships that link DSG and drying method to noodle quality, enabling the targeted design of pre-gelatinized starches for specific textural applications.

To address these research gaps and test the hypothesis, the present study was designed to examine the multi-level molecular structure of potato starch with varying gelatinization degrees followed by either FD or low-temperature OD. This study further extends its scope by incorporating these starch samples into noodles. The integration of potato starch with varying DSG into the wheat flour and the subsequent noodle preparation offer a unique perspective on how these modifications can influence the final product’s characteristics. This work aims to provide novel insights into how the pre-gelatinization process and drying step acts as critical structure-defining operation in starch modification. By elucidating the distinct structure–function relationships imparted by FD and OD, this study will offer a scientific framework for the rational selection and design of pre-gelatinized starches, enabling the targeted development of noodle products and other starch-based foods with customized textural properties.

## 2. Materials and Methods

### 2.1. Raw Materials

Potato starch (PS) (starch content 98.5% on dry weight basis) was purchased from a local market in Beijing, China. Wheat flour (82.4% starch, 14.1% protein, 2.9% fat, and 0.6% ash on dry basis) was purchased from Heng Feng Wheat Flour Co., Ltd. (Bayannur City, Inner Mongolia, China).

### 2.2. Sample Preparation

Partially gelatinized potato starch samples with different degrees of starch gelatinization (DSG) were prepared as described previously [7]. In brief, native potato starch solution (10%, *w*/*w*) was pre-heated at 60 ± 1 °C using a thermostatic water bath for 30 s, 3 min, and 18 min separately. Starch solutions were quickly cooled to room temperature and divided into two parts. One part was subjected to freeze-drying for approximately 48 h in a vacuum freeze dryer (GenesisTM SQ, Virtis, Warminster, PA, USA) and the other part was dried by using an air drying oven (Memmert UF110, Schwabach, Germany) at 40 °C for 24 h. Then, dried samples were ground into powders, sealed in plastic bags and kept in a desiccator at 25 °C. Freeze dried (FD) samples were named as FD-30 s, FD-3 min, FD-18 min, and the oven dried (OD) samples were named as OD-30 s, OD-3 min, and OD-18 min. Native potato starch (NPS) was used as a control sample. For each treatment, three independent batches were prepared from separate weighing and processing runs. All subsequent measurements were performed on these independent batches.

### 2.3. Differential Scanning Calorimetry (DSC)

The thermal properties of partially gelatinized potato starch from freeze drying and air oven drying were analyzed using a DSC analyzer TA Instruments Q2000 (New Castle, DE, USA) according to previously reported methods [11]. Briefly, powder samples (3 mg) were weighed into aluminum pans, mixed with 10 μL of distilled water, and hermetically sealed. After equilibration at room temperature for 2 h, the samples were heated from 20 °C to 100 °C at 5 °C/min. The onset (T_0_), peak (T_p_), and conclusion (T_c_) temperatures, as well as the endothermic enthalpy (ΔH), were obtained from the DSC thermograms using the instrument software. The gelatinization range (R) was calculated as Tc − T0 and peak height index (PHI) as ΔH/(T_p_ − T_0_) [13]. The degree of starch gelatinization (DSG) was calculated using Equation (1).

DSG (%) = (1 − ΔH_sample_/ΔH_native_) × 100% (1)

where ΔH_native_ and ΔH_sample_ represent the enthalpy changes of native and modified starches, respectively [14,15].

### 2.4. X-Ray Diffraction (XRD)

The crystalline structure of the starch samples was determined by XRD using a diffractometer (D8 Advance, Bruker, Germany) according to our previously reported method [11]. Relative crystallinity was calculated using MDI-Jade 6.0 software (Material Data, Inc., Livermore, CA, USA).

### 2.5. Fourier Transform Infrared (FTIR) Spectroscopy

Fourier transform infrared spectroscopy (FTIR) was used to evaluate the short-range ordered structure of the starch samples as previously reported with minor modifications [16]. The starch powder was blended with potassium bromide (KBr) in a ratio of 1:100 (*w*/*w*) and finely ground. The mixture was then pressed into a transparent pellet under hydraulic pressure. FTIR spectra were collected using a spectrometer (Tensor 27, Bruker Opticals Company, Rheinstetten, Germany) over the range of 4000–400 cm^−1^ at a resolution of 4 cm^−1^. Each spectrum was obtained by averaging 64 consecutive scans, using air as the background. The collected spectra were initially subjected to baseline correction using the OMNIC 8.2 software. Subsequently, spectral deconvolution was applied within the range of 1200 to 800 cm^−1^, employing a half-bandwidth of 19 cm^−1^ and an enhancement factor of 1.9. The deconvoluted spectra were used for quantitative analysis by measuring the peak height of relevant absorbance bands relative to the baseline. To characterize the short-range molecular order of starch, the ratios of absorbance intensities at 1047/1022 cm^−1^ and 1022/995 cm^−1^ were calculated.

### 2.6. Pasting Properties

The pasting behavior of the starch samples was evaluated using a Rapid Visco Analyzer (RVA, Perten Instruments, Sydney, Australia) according to the method described previously [17]. Briefly, each starch sample (1.5 g, dry basis) was accurately weighed and dispersed in 25 mL of distilled water. The paddle speed was set at 960 rpm for the first 10 s to ensure uniform dispersion and then maintained at 160 rpm throughout the remaining test. The starch slurry was equilibrated at 50 °C for 1 min, followed by heating to 95 °C at a rate of 12.2 °C/min. After maintaining at 95 °C for 2.5 min, the sample was cooled to 50 °C at 11.8 °C/min and held at this temperature for 2 min. Throughout the heating and cooling cycles, the following pasting parameters were recorded automatically by the instrument software: peak viscosity, trough viscosity, breakdown, final viscosity, setback, pasting temperature, and peak time.

### 2.7. Scanning Electron Microscopy (SEM)

The microstructural features of the partially gelatinized starch powders were examined using a field-emission scanning electron microscope (FE-SEM, SU8010, Hitachi, Tokyo, Japan). The starch samples were directly mounted on aluminum stubs using double-sided conductive adhesive tape. All specimens were subsequently sputter-coated with a thin layer of gold to ensure adequate electrical conductivity. Observations were carried out at an accelerating voltage of 10 kV. Representative micrographs were captured at 500× magnification, with insets showing the same samples at 1000× magnification for detailed surface observation.

### 2.8. Noodle Preparation

Noodles were prepared by incorporating partially gelatinized potato starch samples (as described in Section 2.2) into wheat flour. The starch samples (NPS, FD-30 s, FD-3 min, FD-18 min, OD-30 s, OD-3 min, or OD-18 min) were used to replace 10% of the wheat flour on a dry weight basis (*w*/*w*). The WF control was prepared using 100% wheat flour without any starch substitution. The noodle samples incorporated with NPS, FD-30 s, FD-3 min, FD-18 min, OD-30 s, OD-3 min, and OD-18 min are designated as N-NPS, N-FD-30 s, N-FD-3 min, N-FD-18 min, N-OD-30 s, N-OD-3 min, and N-OD-18 min, respectively. The WF control noodle is designated as N-WF. The moisture content of the dough was adjusted to 35% (*w*/*w*). The noodle-making process was carried out as follows: wheat flour and the pre-gelatinized starch were mixed thoroughly and the mixture was then hydrated with distilled water and kneaded into a dough by using a multifunctional Kenwood Chef (KM300, Kenwood Ltd., Havant, UK) equipped with a dough hook. The resulting dough was rested for 30 min at room temperature to allow water equilibration. It was subsequently subjected to a folding process and a 90° rotation prior to subsequent sheeting. This sequence of folding, rotation, and sheeting was iterated a total of 10 times, after which the dough was allowed an additional 30 min of rest. The resultant dough layer was then sheeted by repeated passes at progressively reduced roll gaps to form a smooth and uniform sheet until achieve the desired thickness (1 mm) [18,19]. Finally, the sheet was cut into strands, and the fresh noodles were dried at room temperature (25 °C).

### 2.9. Color Determination

The expanded dough sheets obtained after rolling in Section 2.8 were used for color detection by using a Chroma meter (CR-400; Konica Minolta, Tokyo, Japan) and represented by the CIE—L*, a* and b* color scale. The parameter L* quantifies luminance on a scale of 0 to 100, where an elevated L* value corresponds to an increased level of brightness. The a* value delineates the chromatic coordinates along the red-green axis, with negative values signifying a green hue and positive values indicating a red tint. Conversely, the b* value represents the chromatic coordinates along the yellow-blue axis, where negative values are indicative of a blue shade, and positive values suggest the presence of yellowness in the product’s color profile [8].

### 2.10. Textural Profile Analysis (TPA)

The dried noodle samples were subjected to a boiling process in a controlled volume of water (700 mL) for 6 min, with a fixed quantity of dried noodles (20 g). The optimal cooking time (6 min) for each noodle formulation was first determined by cooking until the white core disappeared. A uniform cooking time of 6 min was selected for all samples to ensure complete cooking and to allow for valid comparison among formulations. The cooked noodles were rapidly cooled by submerging them in a continuous flow of tap water for a duration of 30 s to arrest the cooking process and lock in the texture prior to undergoing the subsequent testing procedures. The textural profile properties of the cooked noodles were determined using a texture analyzer (TA-XT2i, Stable Microsystems, Godalming, UK) with a P/36R probe. The test parameters were set with a pre-test speed of 2.00 mm/s, a test speed of 0.80 mm/s, and a post-test speed of 2.00 mm/s. Target strain was set to 50.0%, with a trigger force of 0.049 N. TPA was selected because it simulates the dual-bite chewing action of human mastication and provides multiple texture parameters (hardness, springiness, cohesiveness, gumminess, chewiness, resilience) that are directly correlated with sensory perception [20,21].

### 2.11. Statistical Analysis

Mean values and standard deviations were calculated. One-way ANOVA followed by Tukey’s test at the 0.05 significance level was used to evaluate the differences among sample means. The independent samples *t*-test was used for direct comparisons between FD and OD samples at the same pre-heating time. ANOVA was carried out using SPSS 18.0 (SPSS Inc., Chicago, IL, USA), and graphs were plotted using Origin 9. Pearson correlation analysis and plotting were carried out using R (version 3.2.1), with significance levels defined as *p* < 0.05, *p* < 0.01, and *p* < 0.001.

## 3. Results and Discussion

### 3.1. Thermal Properties

**Figure 1:**
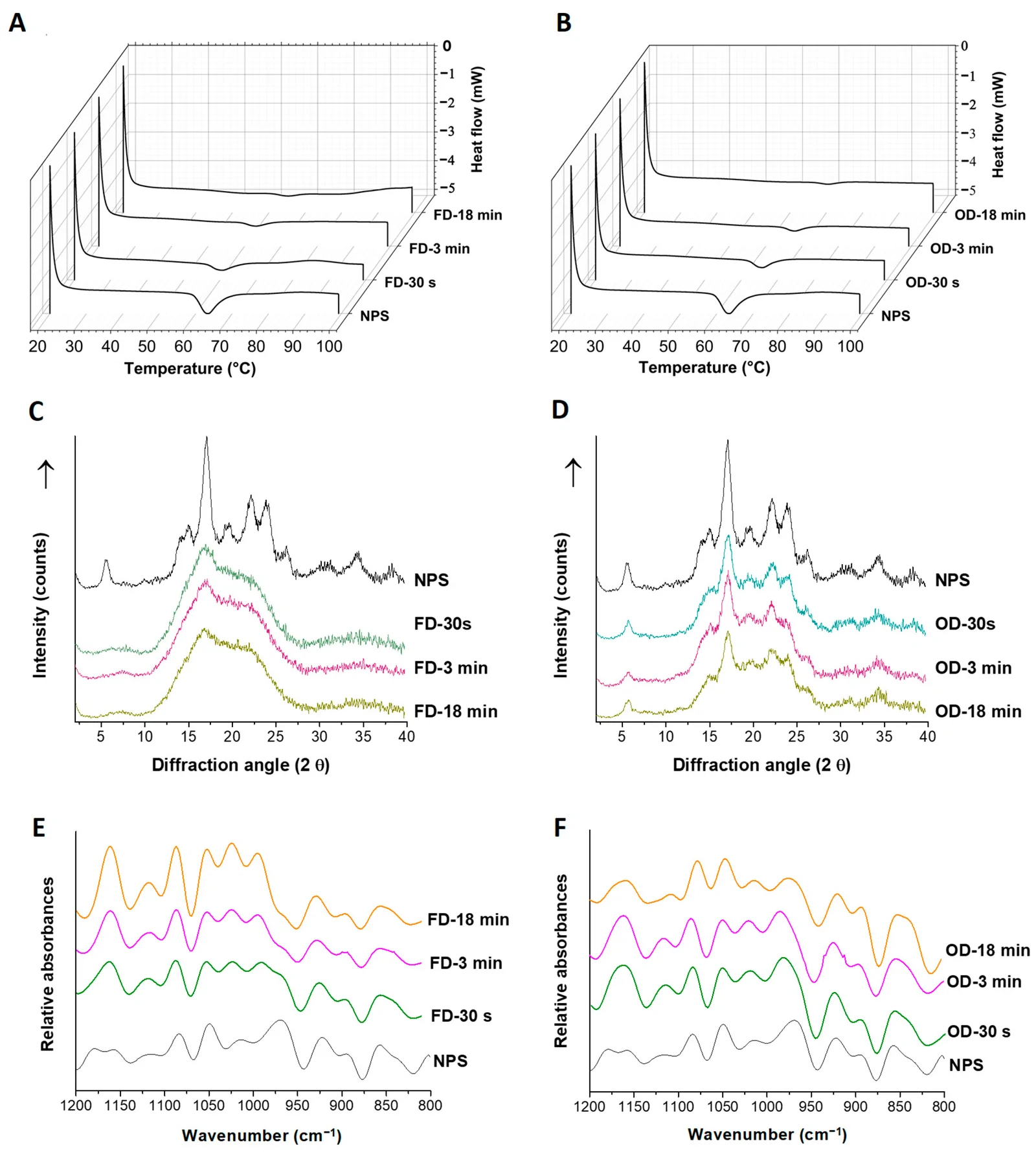
Differential scanning calorimetry (DSC) thermograms (**A**,**B**), X-ray diffraction patterns (**C**,**D**) the upward arrow in the figure indicates the direction of increasing intensity), and FTIR spectra (**E**,**F**) of native and partially gelatinized potato starches with different pre-heating times. (**A**): Freeze-dried (FD) samples; (**B**): Oven-dried (OD) samples. NPS: native potato starch.

The DSC thermograms (Figure 1A,B) clearly showed that the endothermic peaks of both OD and FD samples became smaller and flatter as the pre-heating time increased from 30 s to 18 min. This suggests that the starch granules underwent structural disorganization during the pre-gelatinization process. The DSC parameters in Table 1 showed that the ΔH value for NPS was 17.6 J/g, which was close to previously reported values [22]. Moreover, the ΔH decreased significantly with increasing pre-heating time for both FD and OD series. The corresponding DSG increased accordingly, reaching approximately 90% for both FD-18 min and OD-18 min samples. As is known, the thermal parameters of starch are governed by the structural stability of both its amorphous and crystalline domains, which are in turn influenced by crystal thickness, polymorphic type, and the surface free energy of the crystalline faces [23,24]. In the present study, longer pre-heating time disrupted the crystalline regions and weakened the internal structural integrity of the starch granules. Consequently, less energy was required to achieve complete gelatinization, as reflected by the reduced ΔH and increased DSG. Furthermore, the gelatinization temperatures including T_0_ and T_p_ increased with longer pre-heating times. Similar observations of higher gelatinization temperature in partially gelatinized starch compared to native starch have also been reported in previous studies. [25,26]. This can be attributed to the preferential melting of less stable starch crystals during pre-heating. The remaining crystalline regions after partial gelatinization possess higher thermal stability, thus requiring higher temperatures to melt during subsequent DSC analysis [27]. The PHI, an indicator of the overall integrity of starch crystals, also showed a significant decrease with increasing DSG. The PHI value dropped from 4.48 for NPS to below 0.41 and 0.35 for the FD-18 min and OD-18 min samples, respectively. This significant reduction indicated the loss of molecular order and crystalline structure within the starch granules, aligning with the high DSG values. Moreover, Table 1 indicates that OD samples possessed significantly higher T_0_ and T_p_ than FD samples at the same pre-heating times. This suggests that the slower air-drying process might facilitate additional structural changes in the amorphous regions, leading to a more thermally stable structure compared to the rapid freeze-drying process.

### 3.2. Crystalline Structure

The long-range crystalline structure of the starch samples is displayed in Figure 1C,D. NPS exhibited a characteristic B-type crystalline pattern, with strong diffraction peaks at approximately 5.6°, 17°, 22°, and 24° (2θ), which is consistent with the known crystalline structure of potato starches [28,29]. The intensity of the crystalline peaks gradually diminished with increasing pre-heating time for both FD and OD samples. This indicates a systematic disruption of the crystalline regions within the starch granules during the partial gelatinization process. The longer the starch slurry was pre-heated, the more extensive the breakdown of the double-helical order and the crystalline lamellae, leading to a more amorphous structure [30,31].

**Figure 2:**
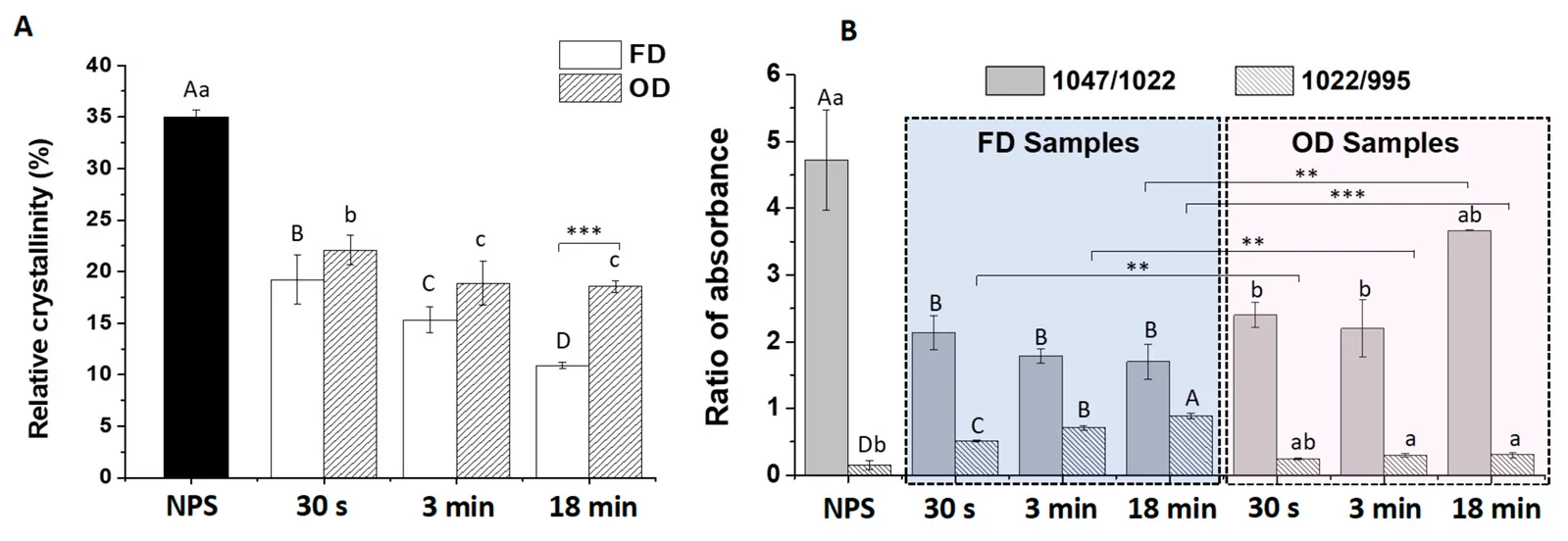
Relative crystallinity (**A**), Ratios of absorbance 1047/1022 cm^−1^ and 1022/995 cm^−1^ (**B**) of native and partially gelatinized potato starches with different pre-heating times (NPS: native potato starch; FD: Freeze-dried samples; OD: Oven-dried samples). Bars represent the mean values, and error bars represent the standard deviation (*n* = 3). ^A,B,C,D^ represent the significant difference of NPS and FD samples (*p* < 0.05); ^a,b,c^ represent the significant difference of NPS and OD samples (*p* < 0.05). * represents the significant difference between FD and OD samples at the same pre-heating time (** *p* < 0.01, *** *p* < 0.001). Statistical differences were determined by one-way ANOVA with Tukey’s test, and independent samples *t*-test for FD–OD comparisons.

The calculated relative crystallinity is shown in Figure 2A; results showed that the relative crystallinity of NPS was the highest among all samples. For both the FD and OD series, the crystallinity decreased significantly with increasing pre-heating time. Clearly, the pre-gelatinization process primarily acts by melting and disorganizing the crystalline domains. Notably, OD samples showed significantly higher relative crystallinity than FD samples at a pre-heating time of 18 min (18.55% vs. 10.90%). This can be attributed to the different drying mechanisms. FD is a rapid process that occurs at low temperatures, effectively freezing the molecular structure in its pre-heated, amorphous state and minimizing molecular rearrangement. In contrast, OD is a gradual, thermal process at 40 °C. This prolonged exposure to a moderate temperature likely facilitated starch retrogradation. As previously reported, retrogradation develops double helices and microcrystalline structures, and therefore promotes the re-formation of crystalline structures within the starch matrix [32]. Consequently, while both methods resulted in a net loss of native crystallinity compared to NPS, the OD process led to the formation of new crystallites, which contributed to the higher measured relative crystallinity in OD samples compared to FD samples.

### 3.3. The Short-Range Ordered Structure

The deconvoluted spectra in the region of 1200–800 cm^−1^ are presented in Figure 1E,F. Results showed that compared to NPS, the relative absorbance of the band at 1047 cm^−1^, associated with the ordered crystalline region, decreased in both FD and OD samples. Conversely, the absorbance of the band at 1022 cm^−1^, related to amorphous starch, became more pronounced. The band at 995 cm^−1^ was mainly caused by the bending vibration of C-OH, corresponding to the hydrogen bond structure formed between the hydroxyl groups of starch macromolecules [33]. Clearly, the absorbance of the band at 995 cm^−1^ of partially gelatinized starch samples decreased compared to NPS. These visual shifts suggest a reduction in molecular order due to the pre-gelatinization treatment.

The ratios of absorbance at 1047/1022 cm^−1^ and 1022/995 cm^−1^, which are suggested to be sensitive to the so-called short-range order, are defined as the degree of ordered double-helical structure and the proportion of amorphous regions, respectively [34,35]. As shown in Figure 2B, for the 1047/1022 cm^−1^ ratio, NPS exhibited the highest value (4.72), confirming its well-ordered native structure. All partially gelatinized samples showed significantly lower ratios, indicating a substantial loss of short-range double-helical order. Moreover, this ratio gradually decreased with increasing pre-heating time in FD samples, demonstrating that longer gelatinization times led to more extensive disruption of molecular order. This is consistent with previous reports that the 1047/1022 ratio of potato starch decreased significantly from the native to the fully gelatinized state [35]. A similar decreasing trend was observed in the OD series for the 30 s and 3 min samples. However, the OD-18 min sample showed higher 1047/1022 ratio compared to other FD and OD samples. This could be due to the formation of new, more thermally stable short-range ordered structures during the prolonged OD drying process, such as those associated with retrograded amylose or recrystallized amylopectin branches. This finding aligns with the significantly higher relative crystallinity observed for OD-18 min in XRD. Conversely, the 1022/995 cm^−1^ ratio of NPS was the lowest, and it increased significantly in both FD and OD samples as pre-heating time increased, confirming the growth of amorphous regions. Moreover, the 1022/995 cm^−1^ ratios of OD samples were significantly lower than those of FD samples, supporting the hypothesis that oven-drying induces partial reordering.

### 3.4. Pasting Properties

**Figure 3:**
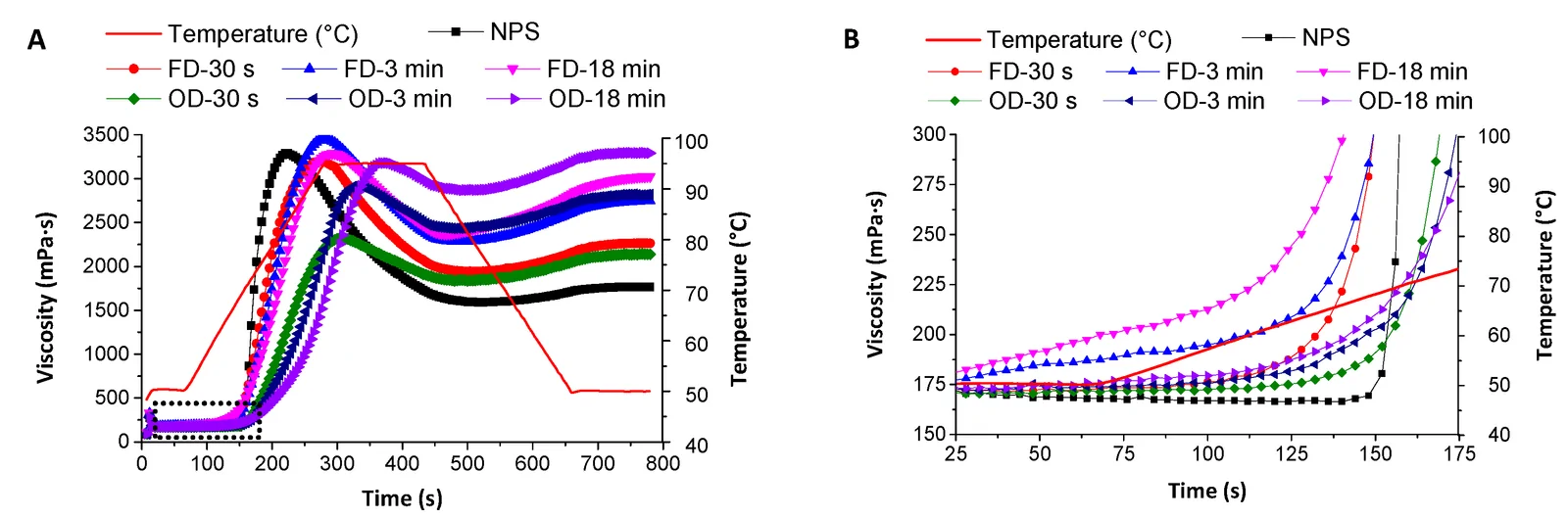
The RVA curves of native and partially gelatinized potato starches with different pre-heating times (NPS: native potato starch; FD: Freeze-dried samples; OD: Oven-dried samples). (**A**): The complete pasting profiles; (**B**): Detailed view of the initial heating phase, corresponding to the area enclosed by the dotted frame in (**A**).

The pasting behavior of the starch samples was evaluated using an RVA as presented in Figure 3. At the beginning of the test (Figure 3B), all pre-gelatinized starch suspensions exhibited a significantly higher initial viscosity compared to NPS. This indicates that the pre-gelatinization process created a certain degree of cold-water swelling capacity and instant viscosity development [36,37]. Notably, the FD series consistently showed higher initial viscosity than their OD counterparts at equivalent pre-heating times. Compared to NPS, all pre-gelatinized samples, particularly the OD samples, exhibited a delayed viscosity development and a reduction in the sharpness of the viscosity peak, indicating a modified granule swelling pattern due to the prior structural disruption.

Table 2 showed the detailed data of the viscosity changes. Results showed that the peak viscosity of the FD series has no significant difference with NPS, while the OD series were significantly lower than that of NPS. Moreover, with longer pre-heating time, the peak viscosity of OD samples increased. The trough viscosity is an indicator of the shear resistance of the starch paste at high temperatures [38]. It was significantly higher in all pre-gelatinized samples compared to NPS, and it increased markedly with longer pre-heating time for both FD and OD series. This indicates that the pre-gelatinization disrupted starch granules formed a more robust paste structure under high-temperature shear, reflecting enhanced paste stability. Breakdown viscosity, which reflects the thermal instability of starch as well as the degree of granule disintegration [38,39], decreased dramatically in all pre-gelatinized samples compared to NPS. This reduction was particularly significant in the OD series. The significantly lower breakdown indicates that OD samples possessed higher stability against shear-thinning at high temperatures. Conversely, the final viscosity and setback, related to gel formation upon cooling, significantly increased in both FD and OD samples compared to NPS. As previously reported, the setback value can be used to determine short-term retrogradation, which is mainly attributed to amylose retrogradation [40]. This indicates that partially gelatinized starch exhibited a higher degree of recrystallization than NPS during retrogradation, particularly in samples subjected to longer pre-heating times. The peak time was consistently delayed in all pre-heated starches compared to NPS, and it increased significantly with increasing DSG. This suggests that the pre-gelatinized granules required a longer heating duration to reach their maximum swelling capacity, likely due to the prior alteration of their crystalline structure. Particularly, the pasting temperature of the FD series was lower than that of NPS and decreased further with increasing DSG. This can be attributed to the extensive disruption of the crystalline regions during pre-gelatinization, which facilitated earlier and easier granular swelling upon reheating. In contrast, the pasting temperature of the OD series was higher than that of NPS and increased progressively with DSG. This suggested that the oven-drying process, which likely promoted retrogradation and recrystallization (as also supported by XRD analysis), created a more thermally stable structure that required higher energy input to initiate pasting.

### 3.5. Morphological Properties

**Figure 4:**
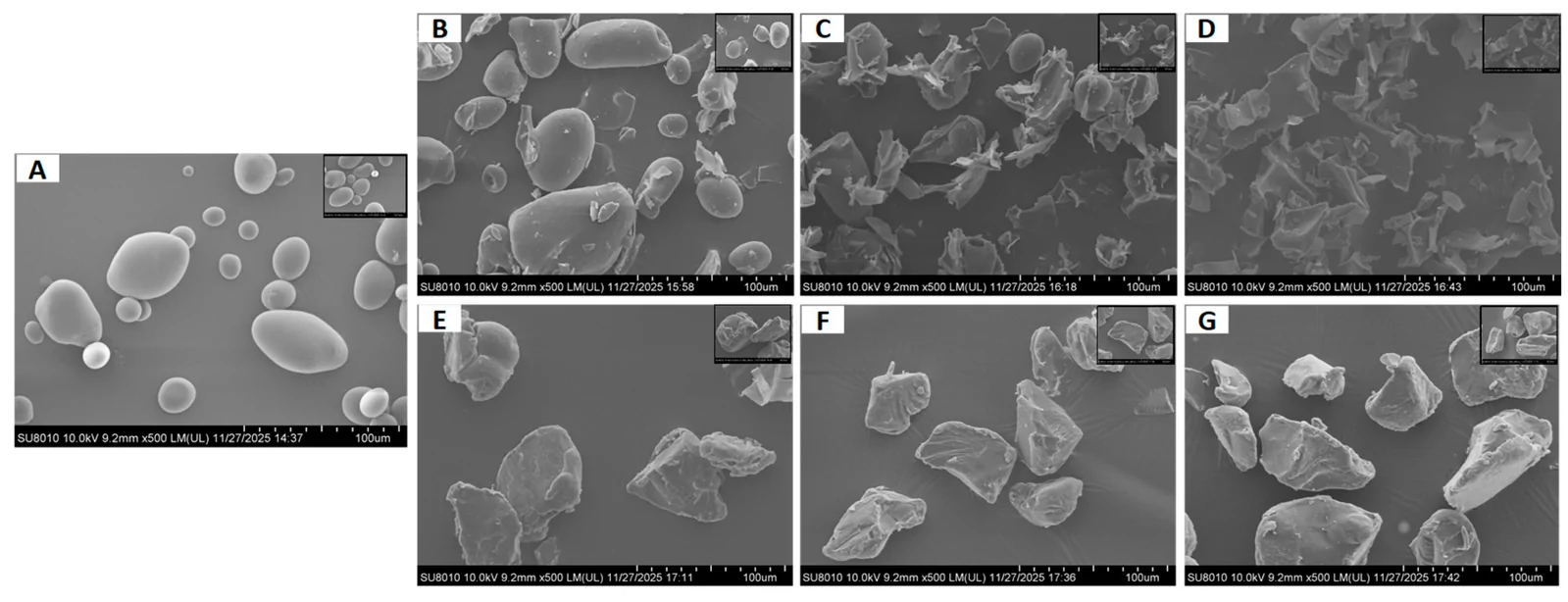
The scanning electron micrographs (**A**–**G**) of native potato starch (**A**) and partially gelatinized potato starches prepared by different drying methods with different pre-heating times ((**B**–**D**): Freeze-dried samples for 30 s, 3 min, and 18 min; (**E**–**G**): Oven-dried samples for 30 s, 3 min, and 18 min).

The morphological changes of starch samples were visualized using SEM, as presented in Figure 4. NPS granules (Figure 4A) exhibited the characteristic oval and spherical shapes with smooth surfaces, which is similar to previous studies [41]. The micrographs of the FD samples showed that after a short pre-heating of 30 s (Figure 4B), granules began to show initial signs of surface roughening and minor swelling, while largely maintaining their original shape. This corresponds to the initial stage of gelatinization where water penetrates the amorphous regions, causing limited swelling [7]. As pre-heating time further increased, the structure disruption became more pronounced, as evidenced by the collapse of starch granules into fragmented flakes. The typical oval or elliptical shape of the starch granules was gradually lost. These extensive morphological changes were consistent with the increased DSG determined by DSC and the low relative crystallinity determined by XRD, confirming that the microscopic structural disruption is a direct consequence of the loss of molecular order.

In contrast, the OD series (Figure 4E–G) displayed a notably different morphological evolution. The partially gelatinized OD starch granules appeared shrunk and collapsed with a denser and less porous appearance. With increasing pre-heating time, the granules lost their individual identities more completely, forming a dense and compacted, block-like morphology. This dense, collapsed structure could be visual evidence of the retrogradation and recrystallization processes that occurred during oven-drying, as inferred from the higher relative crystallinity of OD samples. The thermal energy during drying facilitated the re-association of amylose and short-chain amylopectin, leading to the formation of a new, more thermally stable solid matrix. These distinct microstructural features serve as the foundation for the significant differences observed between FD and OD samples in terms of functional properties, highlighting that the drying step is not merely a moisture removal process but a critical structural-defining operation in the production of modified starches.

### 3.6. Color of Noodle Sheets

**Figure 5:**
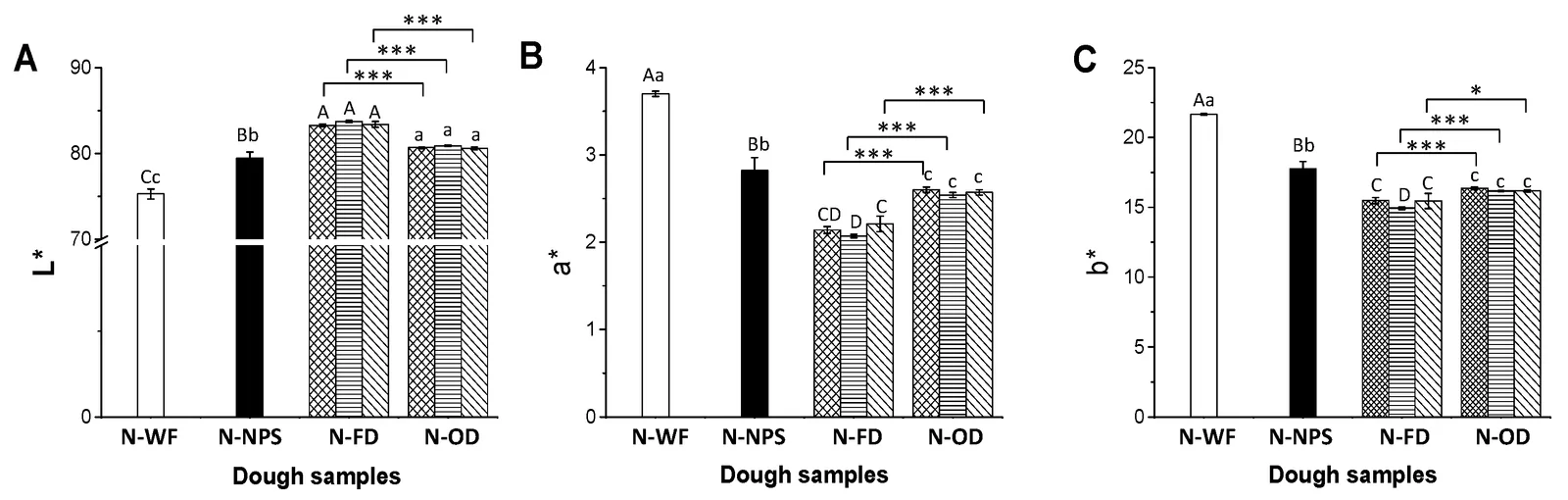
Color parameters of noodle sheets formulated with wheat flour (N-WF), native potato starch (N-NPS), and partially gelatinized potato starches with different pre-heating times. (**A**) Lightness (L* value); (**B**) Redness/Greenness (a* value); (**C**) Yellowness/Blueness (b* value). FD: Freeze-dried samples; OD: Oven-dried samples. In the bar charts, the white bars represent the N-WF control, the black bars represent N-NPS, and the patterned bars represent noodles with different pre-heating times: grid pattern for 30 s, horizontal lines for 3 min, and diagonal lines for 18 min. Bars represent the mean values, and error bars represent the standard deviation (*n* = 3). ^A,B,C,D^ represent the significant difference of N-WF, N-NPS and N-FD samples (*p* < 0.05); ^a,b,c^ represent the significant difference of N-WF, N-NPS and N-OD samples (*p* < 0.05). * represents the significant difference between N-FD and N-OD samples at the same pre-heating time (* *p* < 0.05, *** *p* < 0.001). Statistical differences were determined by one-way ANOVA with Tukey’s test, and independent samples *t*-test for FD–OD comparisons.

The color parameters (L*, a*, b*) of noodle sheets incorporated with different modified starch samples are presented in Figure 5. The visual appearance and color quality of noodles are critical factors influencing consumer acceptance, with a brighter (higher L*) and lighter color generally being preferred [42]. As shown in Figure 5A, the control sample made with WF only exhibited the lowest L* value (75.27). Notably, all noodle sheets containing pre-gelatinized starches (both N-FD and N-OD series) demonstrated an increase in L* value compared to the N-WF control and the N-NPS sample. This indicates that the incorporation of partially gelatinized potato starch effectively brightened the noodle sheets. Consistent with our findings, previous studies have reported that the addition of potato starch can improve noodle transparency and slipperiness [43], as well as color and smoothness [44]. As shown in Figure 5B, noodle sheets of the N-WF control displayed a significantly higher a* value (representing red-green chromaticity) than the N-NPS and the partially gelatinized starch samples. Apparently, the addition of partially gelatinized starch decreased the redness of noodle sheets, and this reduction is desirable for the premium appearance of white salted noodles. Similarly, for the b* value, the N-WF sample also exhibited the highest yellowness. Furthermore, it is worth noting that the drying method also influenced the color parameters. Noodle sheets with added FD starches showed significantly higher L* values and significantly lower a* and b* values than samples in which OD was added. This difference may be partially attributable to the minor enzymatic browning reactions that potentially occurred in OD starches during the drying process. Partially gelatinized potato starch, especially FD starch, could effectively improve the color quality of noodle sheets. By significantly enhancing brightness and reducing undesirable redness and yellowness, partially gelatinized starch can be utilized to develop noodles with a more appealing visual appearance that meets consumer expectations.

### 3.7. Textural Properties of Cooked Noodles

The texture profile of cooked noodles was evaluated using TPA, which has been widely adopted as a standard method for assessing noodle texture and has been shown to be directly correlated with sensory perception [20,21]. The resulting TPA curves and parameters are presented in Figure S1 and Table 3, respectively. The TPA curves provide a visual representation of the textural changes during the two-cycle compression test [45]. The curves for noodles containing pre-gelatinized starches, particularly the OD series, showed distinct shapes compared to the N-WF control and N-NPS sample. Notably, the peak force during the first compression varied significantly among the samples, which is quantified in Table 3. As is shown, the incorporation of pre-gelatinized starches significantly altered the noodle texture, and the effects were highly dependent on both the DSG and the drying method. The N-FD-30 s noodle sample showed a significant increase in hardness, gumminess, and chewiness compared to the N-WF control and N-NPS noodles. This initial increase in firmness could be attributed to the partially gelatinized starch granules acting as rigid fillers and forming a less continuous, more heterogeneous protein–starch matrix [11]. This texture-enhancing effect is consistent with previous findings on modified starch-noodle systems. A previous study reported that incorporating heat–moisture-modified sweet potato starch at 3–9% improved the textural properties of wheat noodles by filling the voids in the gluten network and forming stronger hydrogen bonds with gluten proteins [46]. However, as the DSG increased (N-FD-3 min and N-FD-18 min samples), these parameters decreased progressively. This trend suggests that highly gelatinized starch, with its extensive granular disruption, likely leaches more amylose and forms a softer, more gel-like network within the noodle structure, thereby softening the overall texture. In contrast, the OD series demonstrated a consistently softer texture compared to both the N-WF control and their N-FD counterparts at same pre-heating times. N-OD-30 s and N-OD-3 min noodle samples exhibited significantly lower hardness, gumminess, and chewiness than their N-FD counterparts. This pronounced softening effect can be explained by the more extensive retrogradation and recrystallization that occurred during the slow, thermal air-drying process, as inferred from the XRD analysis. These recrystallized regions might create brittle points or interrupt the continuity of the gluten–starch network, leading to a weaker structure that requires less force to compress and chew. Overall, the incorporation of partially gelatinized FD starch with a low degree of gelatinization significantly improved noodle texture by increasing hardness, gumminess, and chewiness.

### 3.8. Correlation Analysis

**Figure 6:**
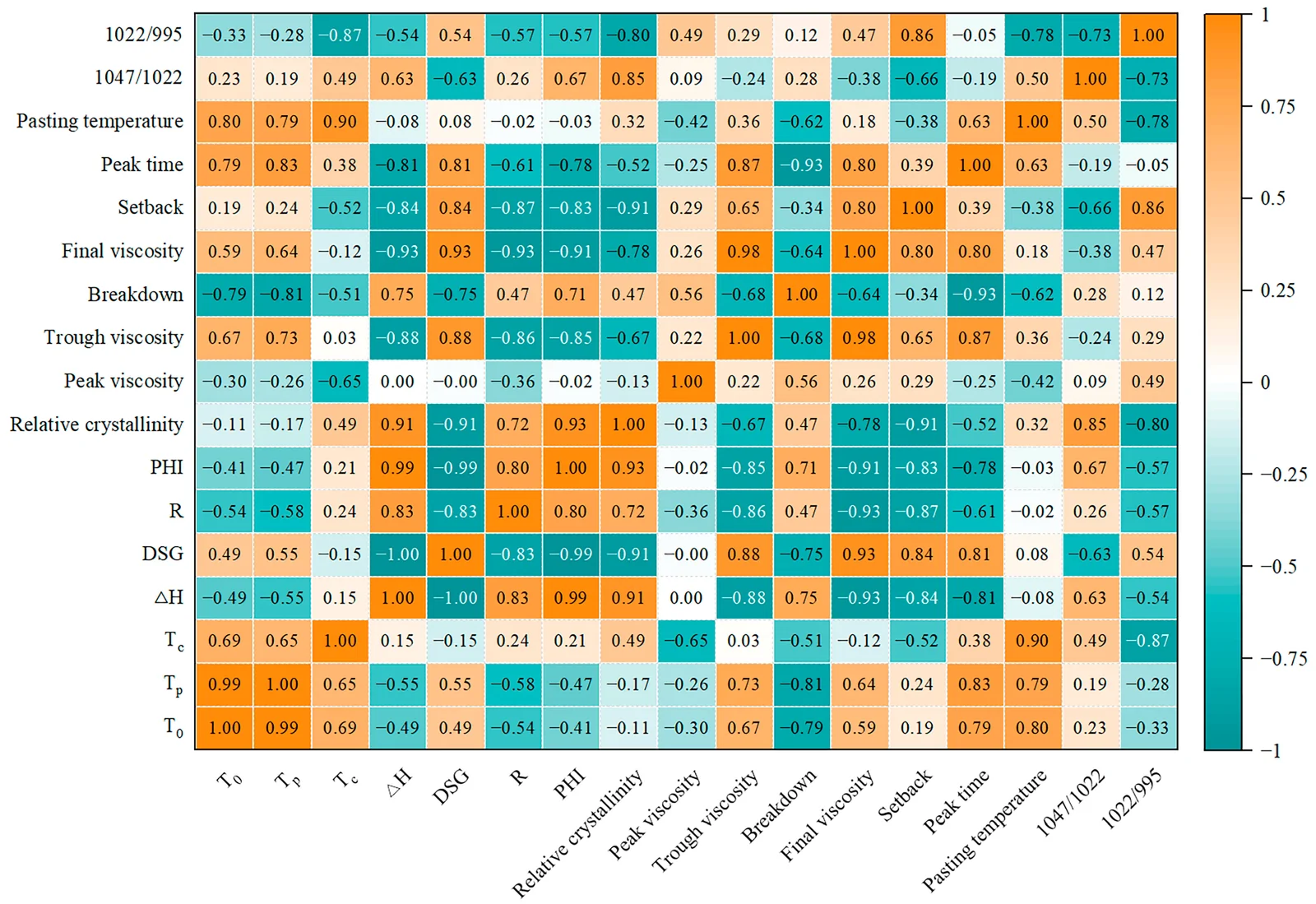
The correlations between the thermal parameters from DSC, relative crystallinity from XRD, viscosity properties from RVA, and the short-range ordered structure from FTIR of starch samples.

To comprehensively elucidate the interrelationships between the structural, thermal, and functional properties of the partially gelatinized potato starches, a Pearson correlation analysis was performed. The resulting correlation matrix is visualized in Figure 6, and the results provided a detailed understanding of how pre-gelatinization and drying methods influence starch functionality. Specifically, a strong positive correlation was observed between the T_c_ from DSC and the pasting temperature from RVA (r = 0.90). This links the completion of the melting endotherm to the onset of macroscopic viscosity development, bridging molecular-level disordering with bulk rheological change. Additionally, relative crystallinity from XRD showed significant negative correlations with DSG (r = −0.91). The absorbance ratio 1047/1022 cm^−1^, indicative of the ordered double-helical content, showed a strong positive correlation with the relative crystallinity. On the contrary, the absorbance ratio 1022/995 cm^−1^, often associated with the amorphous character, displayed a negative correlation with the relative crystallinity. This indicated that higher DSG of partially gelatinized starch leads to the loss of its short-range ordered structure and long-range structure (relative crystallinity). Moreover, trough viscosity and final viscosity were significantly correlated (r = 0.98), suggesting that the paste stability established at high temperature directly dictates the final gel viscosity upon cooling. Both these viscosity parameters showed strong positive correlations with DSG. This clearly demonstrates that higher DSG leads to the formation of a paste with greater stability during heating (higher trough viscosity) and a stronger gel network after cooling (higher final viscosity and setback). Higher DSG delays the pasting temperature, enhances hot-paste stability, and promotes cold-set gelation. The strong correlations between DSC, XRD, RVA, and FTIR parameters validate the complementary nature of these analytical techniques and provide a solid foundation for predicting the functional performance of pre-gelatinized starch based on its fundamental structural and thermal characteristics.

## 4. Conclusions

In this study, the effects of DSG and drying methods on the multi-scale structural properties of potato starch and the resultant noodle quality were systematically evaluated. Pre-gelatinization disrupted the short- and long-range molecular order of starch, while the drying method critically defined the final structural state. OD partially gelatinized starch showed higher relative crystallinity than FD samples, which was caused by the formation of new crystalline structures during OD process. Higher viscosities were observed in partially gelatinized starch samples, compared to NPS, due to the extensive disruption of the crystalline regions during pre-gelatinization. FD samples exhibited a collapse of starch granules into fragmented flakes, whereas OD samples displayed a dense and compacted, block-like morphology. In noodle application, low-DSG FD starch significantly enhanced noodle hardness, gumminess, and chewiness, whereas OD starch consistently yielded softer noodles with reduced hardness. These results suggest that FD starch with low DSG may be more suitable for producing firmer noodles, while OD starch with higher DSG may be preferred for producing softer noodles. However, it should be noted that these implications are based solely on instrumental texture measurements, and further sensory evaluation is needed to confirm consumer acceptance. The structure–function relationships established in this study provide a scientific framework for the rational design of pre-gelatinized starches tailored for specific food applications. Further studies are needed to elucidate the molecular mechanisms underlying the starch–gluten interactions modulated by different drying methods, particularly in complex food systems.

## Figures and Tables

**Table 1:** Thermal parameters of native and partially gelatinized potato starches with different pre-heating times (NPS: native potato starch; FD: Freeze-dried samples; OD: Oven-dried samples).

	T_0_ (°C)	T_p_ (°C)	T_c_ (°C)	△H (J/g)	DSG (%)	R	PHI
NPS	59.43 ± 0.16 ^Bd^	63.36 ± 0.17 ^Bd^	78.08 ± 2.33 ^Aa^	17.6 ± 0.54 ^Aa^	0	18.65 ± 2.19 ^Aa^	4.48 ± 0.14 ^Aa^
FD-30 s	55.82 ± 0.12 ^D^**	60.46 ± 0.29 ^C^*	74.04 ± 0.96 ^A^	8.71 ± 0.12 ^B^*	50.50 ± 0.68 ^C^*	18.23 ± 1.08 ^A^	1.88 ± 0.09 ^B^
FD-3 min	58.29 ± 0.20 ^C^*	63.29 ± 0.18 ^B^**	73.45 ± 1.70 ^A^	5.05 ± 0.43 ^C^	71.33 ± 2.43 ^B^	15.16 ± 1.90 ^AB^	1.01 ± 0.08 ^C^
FD-18 min	61.07 ± 0.07 ^A^*	65.22 ± 0.40 ^A^**	73.47 ± 1.52 ^A^	1.69 ± 0.13 ^D^	90.39 ± 0.76 ^A^	12.40 ± 1.45 ^B^	0.41 ± 0.07 ^D^
OD-30 s	61.61 ± 0.23 ^c^**	65.75 ± 0.05 ^c^*	79.56 ± 0.09 ^a^	7.60 ± 0.15 ^b^*	56.85 ± 0.88 ^c^*	17.95 ± 0.32 ^a^	1.84 ± 0.09 ^b^
OD-3 min	64.06 ± 0.40 ^b^*	67.97 ± 0.29 ^b^**	80.20 ± 0.28 ^a^	4.42 ± 0.13 ^c^	74.87 ± 0.74 ^b^	16.14 ± 0.68 ^ab^	1.15 ± 0.24 ^c^
OD-18 min	66.14 ± 0.49 ^a^*	70.43 ± 0.40 ^a^**	78.85 ± 0.13 ^a^	1.52 ± 0.07 ^d^	91.39 ± 0.41 ^a^	12.71 ± 0.62 ^b^	0.35 ± 0.01 ^d^

Data are means ± SD. ^A,B,C,D^. represent the significant difference of NPS and FD samples in column (*p* < 0.05); ^a,b,c,d^ represent the significant difference of NPS and OD samples in column (*p* < 0.05). * represents the significant difference between FD and OD samples at the same pre-heating time (* *p* < 0.05, ** *p* < 0.01). T_0_: Onset temperature; T_p_: peak temperature; T_c_: conclusion temperature; ΔH: endothermic enthalpy; DSG: degree of starch gelatinization; R: Gelatinization range; PHI: peak height index. Statistical differences were determined by one-way ANOVA with Tukey’s test, and independent samples *t*-test for FD–OD comparisons.

**Table 2:** RVA parameters of native and partially gelatinized potato starches with different pre-heating times (NPS: native potato starch; FD: Freeze-dried samples; OD: Oven-dried samples).

	Peak Viscosity (mPa·s)	Trough Viscosity (mPa·s)	Breakdown (mPa·s)	Final Viscosity (mPa·s)	Setback (mPa·s)	Peak Time (min)	Pasting Temperature (°C)
NPS	3291.50 ± 122.33 ^Aa^	1590.00 ± 36.77 ^Dd^	1701.50 ± 85.56 ^Aa^	1765.00 ± 15.56 ^Dd^	175.00 ± 21.21 ^Dc^	3.73 ± 0.00 ^Cd^	68.75 ± 0.07 ^Ad^
FD-30 s	3199.00 ± 123.04 ^A^	1940.50 ± 36.06 ^C^	1258.50 ± 86.97 ^B^*	2263.50 ± 28.99 ^C^	323.00 ± 7.07 ^C^	4.60 ± 0.10 ^B^	66.10 ± 0.14 ^B^*
FD-3 min	3456.50 ± 139.30 ^A^	2282.50 ± 27.58 ^B^	1174.00 ± 111.72 ^B^*	2746.50 ± 28.99 ^B^	464.00 ± 1.41 ^B^	4.67 ± 0.09 ^B^	66.55 ± 0.57 ^B^
FD-18 min	3286.50 ± 7.78 ^A^	2367.50 ± 4.95 ^A^**	919.00 ± 2.83 ^C^	3025.00 ± 9.90 ^A^*	657.50 ± 4.95 ^A^*	4.87 ± 0.00 ^A^*	64.88 ± 0.60 ^C^*
OD-30 s	2323.50 ± 3.54 ^c^	1837.50 ± 21.92 ^c^	486.00 ± 18.38 ^b^*	2140.00 ± 15.56 ^c^	302.50 ± 6.36 ^b^	5.03 ± 0.14 ^c^	69.88 ± 0.53 ^c^*
OD-3 min	2920.00 ± 115.97 ^b^	2432.00 ± 65.05 ^b^	488.00 ± 50.91 ^b^*	2817.50 ± 51.62 ^b^	385.50 ± 13.44 ^a^	5.54 ± 0.09 ^b^	71.15 ± 0.00 ^b^
OD-18 min	3190.00 ± 138.59 ^ab^	2865.00 ± 14.14 ^a^**	325.00 ± 124.45 ^b^	3289.50 ± 0.71 ^a^*	424.50 ± 14.85 ^a^*	6.23 ± 0.14 ^a^*	72.73 ± 0.04 ^a^*

Data are means ± SD. ^A,B,C,D^. represent the significant difference of NPS and FD samples in column (*p* < 0.05); ^a,b,c,d^ represent the significant difference of NPS and OD samples in column (*p* < 0.05). * represents the significant difference between FD and OD samples at the same pre-heating time (* *p* < 0.05, ** *p* < 0.01). Statistical differences were determined by one-way ANOVA with Tukey’s test, and independent samples *t*-test for FD–OD comparisons.

**Table 3:** Texture profile analysis (TPA) parameters of cooked noodles incorporated with native and partially gelatinized potato starches.

	Hardness (N)	Adhesiveness (N.s)	Resilience (%)	Cohesion	Springiness (%)	Gumminess	Chewiness
N-WF	33.36 ± 5.45 ^ABa^	−1.41 ± 0.62 ^Ab^	50.66 ± 1.61 ^ABbc^	0.85 ± 0.02 ^ABa^	93.68 ± 3.30 ^Aa^	28.36 ± 4.40 ^ABa^	26.52 ± 3.98 ^ABa^
N-NPS	31.61 ± 3.52 ^BCa^	−1.30 ± 0.50 ^Ab^	49.18 ± 2.48 ^Bc^	0.83 ± 0.02 ^Bb^	92.89 ± 2.86 ^Aa^	26.27 ± 2.35 ^BCa^	24.42 ± 2.54 ^BCab^
N-FD-30 s	38.66 ± 7.83 ^A^**	−3.48 ± 3.96 ^B^	50.73 ± 1.34 ^AB^	0.84 ± 0.02 ^AB^	93.44 ± 1.59 ^A^	32.57 ± 6.48 ^A^**	30.37 ± 5.62 ^A^***
N-FD-3 min	31.54 ± 3.36 ^BC^***	−1.18 ± 0.36 ^A^***	50.21 ± 2.24 ^AB^**	0.85 ± 0.02 ^AB^	93.12 ± 2.43 ^A^	26.74 ± 2.42 ^BC^***	24.91 ± 2.46 ^BC^***
N-FD-18 min	27.34 ± 5.33 ^C^	−1.43 ± 0.83 ^A^*	52.01 ± 2.11 ^A^	0.86 ± 0.01 ^A^	91.45 ± 3.12 ^A^	23.47 ± 4.35 ^C^	21.51 ± 4.30 ^C^
N-OD-30 s	25.01 ± 3.97 ^b^**	−0.78 ± 0.19 ^a^	52.33 ± 2.27 ^ab^	0.85 ± 0.02 ^a^	91.07 ± 4.56 ^a^	21.24 ± 2.91 ^b^**	19.43 ± 3.34 ^bc^***
N-OD-3 min	22.45 ± 5.11 ^b^***	−0.54 ± 0.22 ^a^***	53.57 ± 2.85 ^a^**	0.86 ± 0.02 ^a^	105.62 ± 47.52 ^a^	19.32 ± 3.95 ^b^***	20.49 ± 10.33 ^bc^***
N-OD-18 min	23.69 ± 4.25 ^b^	−0.61 ± 0.28 ^a^*	52.34 ± 1.95 ^ab^	0.85 ± 0.01 ^a^	91.47 ± 3.58 ^a^	20.20 ± 3.43 ^b^	18.47 ± 3.20 ^c^

Data are means ± SD. ^A,B,C^ represent the significant difference of N-WF, N-NPS and N-FD noodle samples in column (*p* < 0.05); ^a,b,c^ represent the significant difference of N-WF, N-NPS and N-OD noodle samples in column (*p* < 0.05). * represents the significant difference between FD and OD samples at the same pre-heating time (* *p* < 0.05, ** *p* < 0.01, *** *p* < 0.001). Statistical differences were determined by one-way ANOVA with Tukey’s test, and independent samples *t*-test for FD–OD comparisons.

## Data Availability

The original contributions presented in this study are included in the article/Supplementary Materials. Further inquiries can be directed to the corresponding author.

## Supplementary Materials

The following supporting information can be downloaded at: https://www.mdpi.com/article/10.3390/foods15203752/s1, Figure S1: Typical texture profile analysis (TPA) force-time curves of cooked noodles: Wheat flour noodle (N-WF) noodles; noodles with native potato starch (N-NPS); noodles with freeze-dried (N-FD) partially gelatinized starch; noodles with oven-dried (N-OD) partially gelatinized starch.
