# Supplementary material for: Comparative Analysis of Pre-Gelatinized Potato Starch Prepared by Different Drying Methods and Its Influence on Noodle Quality
Source: Foods. 2026 Oct 21;15(20):3752. doi: 10.3390/foods15203752 (PMC13704128; doi:10.3390/foods15203752)
Supplement: Supplementary file 1 [file foods-15-03752-s001.zip › foods-4607455-supplementary.pdf]

Supplementary data

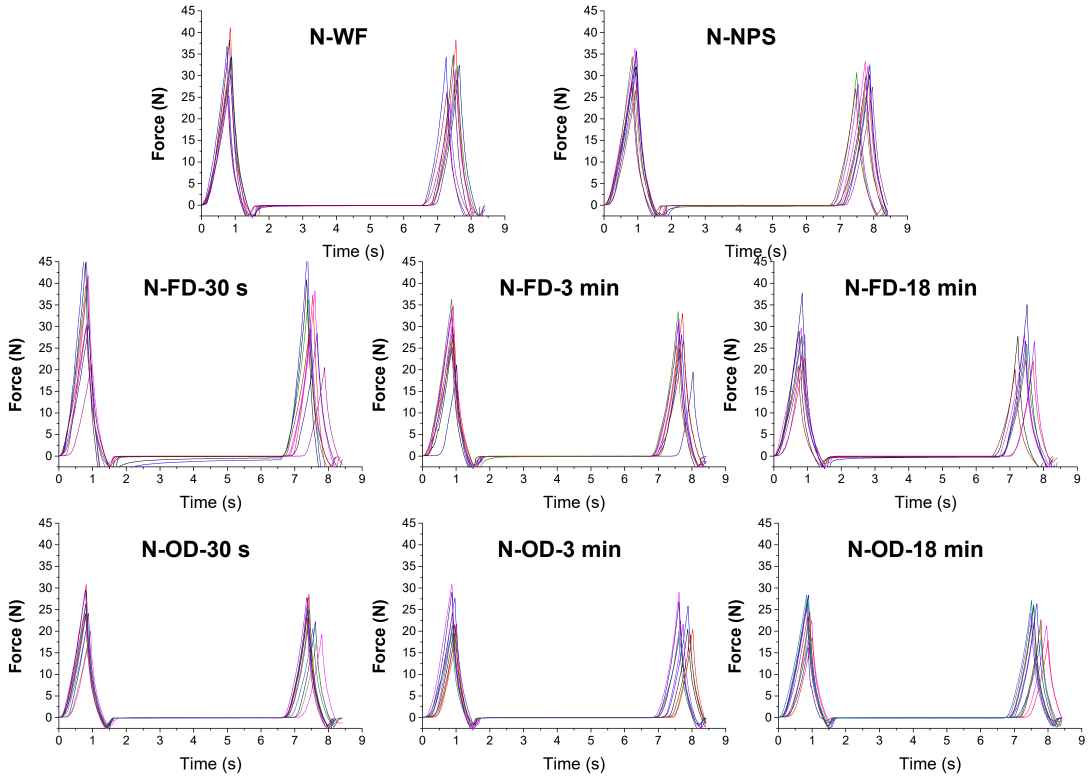

Figure S1. Typical texture profile analysis (TPA) force-time curves of cooked noodles: Wheat flour noodle (N-WF) noodles; noodles with native potato starch (N-NPS); noodles with freeze-dried (N-FD) partially gelatinized starch; noodles with oven-dried (N-OD) partially gelatinized starch.
